# Neonatal outcomes following different ovarian stimulation protocols in fresh single embryo transfer

**DOI:** 10.1038/s41598-019-38724-2

**Published:** 2019-02-28

**Authors:** Seung Chik Jwa, Akira Nakashima, Akira Kuwahara, Kazuki Saito, Minoru Irahara, Tetsuro Sakumoto, Osamu Ishihara, Hidekazu Saito

**Affiliations:** 1 Department of Obstetrics and Gynecology, Saitama Medical University, Saitama, Japan; 2 Sora no Mori Clinic, Okinawa, Japan; 3 Department of Obstetrics and Gynecology, Graduate School of Biomedical Sciences, Tokushima University, Tokushima, Japan; 4 Department of Comprehensive Reproductive Medicine, Graduate School, Tokyo Medical and Dental University, Tokyo, Japan; 5 Division of Reproductive Medicine, Center of Maternal–Fetal, Neonatal and Reproductive Medicine, National Center for Child Health and Development, Tokyo, Japan

## Abstract

Previous studies suggested ovarian stimulation was associated with lower birth weight and higher risk of preterm delivery (PTD) from fresh embryo transfers (ETs). However, whether the increased risk differs between distinct ovarian stimulation protocols remains unknown. A retrospective cohort study of 38,220 singleton deliveries after fresh single ETs from 2007 to 2013 was conducted. Main outcomes were birth weight and gestational length. Compared with the natural cycle, all ovarian stimulation protocols were associated with a significantly increased risk for PTD, low birth weight (LBW) and small for gestational age (SGA). In subgroup analysis of maternal age under 35 years, luteal support using progesterone, and early cleavage ETs, the significant associations remained for LBW and SGA in gonadotropin-releasing hormone (GnRH) antagonist protocol and for LBW in GnRH agonist protocol. Ovarian stimulation using clomiphene citrate (CC) had the highest increased risks for LBW (Adjusted odds ratio [AOR], 1.58, 95% confidence interval [95% CI], 1.43−1.73) and SGA (AOR, 1.65, 95% CI, 1.50−1.82) compared with natural cycles, and was further associated with PTD and cesarean section. These findings suggest ovarian stimulation was associated with lower birth weight, and CC may have adverse effect on neonatal outcomes in fresh cycles.

## Introduction

Since the first baby was born after *in vitro* fertilization (IVF) in the United Kingdom in 1978^1^, assisted reproductive technology (ART), including IVF and embryo transfers (ETs), has been widely used for infertility treatment worldwide. The International Committee for Monitoring Assisted Reproductive Technologies reported that more than one million babies were born after ART between 2008 and 2010^2^. An increased use of ART is also found in Japan, with 51,001 babies reportedly born following ART in 2015, accounting for approximately 1 in 19.7 births^3^.

Despite the dramatic increase in pregnancies following ART, the safety of these techniques continues to be a matter of concern. Observational studies have suggested that babies born after fresh ETs are associated with adverse perinatal outcomes, such as lower birth weight, preterm delivery (PTD) and perinatal deaths, compared with frozen ETs^4^. Recent randomized controlled trials (RCTs) demonstrated that babies born after fresh ETs were significantly smaller than babies born after frozen ETs for women with or without polycystic ovary syndrome^5,6^. Although various processes and procedures related to ART, such as multiple gestations and vanishing twins following multiple embryo transfers, can carry a risk for these adverse perinatal outcomes, the hormonal environment caused by ovarian stimulation in fresh ET may also influence these perinatal outcomes^7–9^.

Ovarian stimulation plays a vital part in ART, allowing the retrieval of multiple oocytes and increasing the success rate of live births per fresh cycles. Several ovarian stimulation protocols have been developed to optimize the number of oocytes retrieved and minimize risks of complications, such as using gonadotropin-releasing hormone (GnRH) agonist^10^, GnRH antagonist^11,12^, and mild ovarian stimulation using clomiphene citrate (CC) or natural cycle IVF (natural cycle)^13,14^. It was suggested that children born following ovarian stimulation may exhibit lower birth weight and higher risk of PTD compared with those following natural cycles^7,8^. Whether the increased risk differs between distinct ovarian stimulation protocols used in fresh ET cycles remains unknown.

We investigated whether ovarian stimulation protocols were associated with birth weight and gestational length in singletons born after fresh single ETs using a nationally-representative ART sample from Japan.

## Results

### Baseline characteristics

Baseline characteristics stratified by ovarian stimulation protocols are shown in Table 1. The sample included natural (n = 4058), CC (n = 4715), CC + gonadotropin (n = 5443), GnRH agonist (n = 16,566) and GnRH antagonist (n = 7483) protocols. Mean maternal age was higher for the CC and natural cycle cohorts, in which 15.4% and 12.6%, respectively, were more than 40 years of age. The proportion of cases with tubal factor/endometriosis was highest for the GnRH agonist protocol, while unexplained infertility was highest in the natural cycle and CC cohorts. The number of oocytes retrieved was highest for the GnRH agonist protocol, followed by the GnRH antagonist protocol, in which approximately 30% of cases had retrieved more than 10 oocytes. For the ovarian stimulation protocols using GnRH agonist or antagonist, over 40% of each cohort used blastocyst ET, while early cleavage ET dominated for the natural cycle, CC and CC + gonadotropin protocols. For luteal support, progesterone was most frequently used in natural cycle and CC, while estrogen + progesterone was used frequently in GnRH agonist and antagonist protocols.

**Table 1:** Baseline characteristics of sample population stratified by ovarian stimulation protocols (n = 38,220)^a^.

Characteristics	Natural cycle (n = 4058)	Clomiphene alone (n = 4715)	Clomiphene + gonadotropin (n = 5443)	GnRH agonist (n = 16566)	GnRH antagonist (n = 7438)	P value^b^
Maternal age, (year)	35.3 (3.6)	35.8 (3.7)	34.7 (3.9)	34.2 (3.7)	34.7 (3.9)	<0.001
<30	253 (6.2)	250 (5.3)	533 (9.8)	1808 (10.9)	697 (9.4)	<0.001
30–34	1367 (33.7)	1421 (30.1)	2010 (36.9)	6737 (40.7)	2774 (37.3)	
35–39	1927 (47.5)	2318 (49.2)	2333 (42.9)	6860 (41.4)	3172 (42.7)	
≥40	511 (12.6)	726 (15.4)	566 (10.4)	1161 (7.0)	795 (10.7)	
Infertility diagnosis^c^						
Tubal factor	494 (12.2)	542 (11.5)	789 (14.5)	3721 (22.5)	1340 (18.0)	<0.001
Endometriosis	174 (4.3)	235 (5.0)	365 (6.7)	1718 (10.4)	658 (8.9)	<0.001
Antisperm antibody	11 (0.27)	9 (0.19)	42 (0.77)	187 (1.1)	74 (0.99)	<0.001
Male factor	656 (16.2)	895 (19.0)	1503 (27.6)	5363 (32.4)	2666 (35.8)	<0.001
Unexplained	2684 (66.1)	3007 (63.8)	2602 (47.8)	5864 (35.4)	2600 (35.0)	<0.001
Others	235 (5.7)	212 (4.5)	729 (13.4)	1666 (10.1)	1084 (14.6)	<0.001
Number of oocytes retrieved	1.2 (0.69)	2.0 (1.2)	3.9 (2.8)	8.6 (5.0)	7.6 (4.8)	<0.001
1	3586 (88.4)	1752 (37.2)	815 (15.0)	455 (2.8)	399 (5.3)	<0.001
2–3	433 (10.7)	2543 (53.9)	2184 (40.1)	1807 (10.9)	1102 (14.8)	
4–9	34 (0.84)	406 (8.6)	2181 (40.1)	8172 (49.3)	3762 (50.6)	
≥10	5 (0.12)	14 (0.30)	263 (4.8)	6132 (37.0)	2175 (29.2)	
Fertilization method						
IVF	2160 (53.2)	2303 (48.8)	2792 (51.3)	8234 (49.7)	2987 (40.2)	<0.001
ICSI	1847 (45.5)	2102 (44.6)	2196 (40.4)	6219 (37.5)	3534 (47.5)	
Split (IVF + ICSI)	51 (1.3)	310 (6.6)	455 (8.4)	2113 (12.8)	917 (12.3)	
Embryo stage at transfer						
Early cleavage	3542 (87.3)	4509 (95.6)	4303 (79.1)	9044 (54.6)	4323 (58.1)	<0.001
Blastocyst	516 (12.7)	206 (4.4)	1140 (20.9)	7522 (45.4)	3115 (41.9)	
Luteal support^c^						
None	358 (8.8)	161 (3.4)	290 (5.3)	160 (0.97)	78 (1.1)	<0.001
Progesterone	3326 (82.0)	3466 (73.5)	2563 (47.1)	4828 (29.1)	2061 (27.7)	<0.001
hCG	58 (1.4)	758 (16.1)	199 (3.7)	1607 (9.7)	337 (4.5)	<0.001
Progesterone + hCG	196 (4.8)	182 (3.9)	1013 (18.6)	4605 (27.8)	2034 (27.4)	<0.001
Estrogen + Progesterone	126 (3.1)	164 (3.5)	1357 (24.9)	5813 (35.1)	3247 (43.7)	<0.001
Others	15 (0.37)	24 (0.51)	200 (3.7)	1013 (6.1)	183 (2.5)	<0.001
Year^d^						
2007	343 (9.6)	535 (15.0)	532 (14.9)	1625 (45.5)	533 (14.9)	<0.001
2008	342 (7.7)	470 (10.6)	622 (14.0)	2259 (50.7)	764 (17.1)	
2009	500 (8.8)	614 (10.8)	790 (13.9)	2688 (47.2)	1100 (19.3)	
2010	537 (9.4)	716 (12.5)	808 (14.1)	2457 (42.8)	1223 (21.3)	
2011	624 (10.4)	692 (11.5)	832 (13.9)	2524 (42.1)	1326 (22.1)	
2012	876 (13.7)	842 (13.2)	887 (13.9)	2526 (39.6)	1251 (19.6)	
2013	836 (13.1)	846 (13.3)	972 (15.2)	2487 (39.0)	1241 (19.5)	

^a^Data are presented as mean (SD) for continuous variables and n (%) for dichotomous variables.

^b^P values were assessed with the use of χ^2^ or one-way analysis of variance.

^c^Multiple answers were allowed.

^d^Percentages for rows for the purpose of comparison.

ART, assisted reproductive technology; ICSI, intracytoplasmic sperm injection; IVF, *in vitro* fertilization

### Neonatal outcomes according to ovarian stimulation protocols

Pregnancy and neonatal outcomes stratified by ovarian stimulation protocols are shown in Table 2. For the natural cycle, term deliveries were the most frequent (90.1%), while PTD and very PTD (VPTD) were the least frequent (5.4% and 0.89%, respectively). Similarly, low birth weight (LBW) and very LBW (VLBW) were least frequent (8.2% and 0.69%, respectively) in the natural cycle cohort. The proportion of small for gestational age (SGA) was highest in the CC + gonadotropin cohort (9.5%), whereas the natural cycle cohort had the significantly lowest frequency (5.4%) of SGA. Cesarean section (CS) was most frequent in the CC cohort (31.0%).

**Table 2:** Pregnancy and neonatal outcomes stratified by ovarian stimulation protocols^a^.

Outcomes	Natural cycle (n = 4058)	Clomiphene alone (n = 4715)	Clomiphene + gonadotropin (n = 5443)	GnRH agonist (n = 16566)	GnRH antagonist (n = 7438)	P value^b^
**Pregnancy outcomes**						
Mode of delivery						
Vaginal	2732 (67.2)	2963 (62.8)	3357 (61.7)	10791(65.1)	4691 (63.1)	<0.001
CS	1100 (27.1)	1463 (31.0)	1501 (27.6)	4294 (25.9)	2022 (27.2)	
Unknown	226 (5.6)	289 (6.1)	585 (10.8)	1481 (8.9)	725 (9.8)	
**Neonatal outcomes**						
Gestational weeks at delivery, (weeks)	38.8 (1.7)	38.6 (1.9)	38.6 (2.0)	38.6 (1.9)	38.6 (1.9)	<0.001
≧37	3658 (90.1)	4185 (88.8)	4467 (82.1)	13844 (83.6)	6127 (82.4)	<0.001
32–36	181 (4.5)	287 (6.1)	281 (5.2)	987 (6.0)	435 (5.9)	
<32	36 (0.89)	50 (1.1)	75 (1.4)	157 (0.95)	80 (1.1)	
Unknown	183 (4.5)	193 (4.1)	620 (11.4)	1578 (9.5)	796 (10.7)	
Birthweight, (g)	3008 (426)	2928 (476)	2927 (487)	2950 (451)	2954 (455)	<0.001
≧2500	3569 (87.8)	3945 (83.4)	4276 (78.6)	13354 (80.6)	5935 (79.8)	<0.001
1500–2499	303 (7.5)	545 (11.6)	585 (10.8)	1634 (9.9)	718 (9.7)	
<1500	28 (0.69)	60 (1.3)	93 (1.7)	168 (1.0)	78 (1.1)	
Unknown	158 (3.9)	165 (3.5)	489 (9.0)	1410 (8.5)	707 (9.5)	
Sex of neonates						
Male	1962 (48.4)	2300 (48.8)	2494 (45.8)	7877 (47.6)	3453 (46.4)	0.19
Female	1939 (47.8)	2253 (47.8)	2465 (45.3)	7323 (44.2)	3278 (44.1)	
Unknown	157 (3.9)	162 (3.4)	484 (8.9)	1366 (8.3)	707 (9.5)	
	(n = 3851)	(n = 4508)	(n = 4764)	(n = 14823)	(n = 6571)	
SGA^c^	208 (5.4)	397 (8.8)	450 (9.5)	998 (6.7)	455 (6.9)	<0.001
LGA^c^	380 (9.9)	400 (8.9)	411 (8.6)	1350 (9.1)	619 (9.4)	0.29

^a^Data are presented as mean (SD) for continuous variables and n (%) for dichotomous variables.

^b^P values were assessed with the use of χ^2^ test excluding missing values or one-way analysis of variance.

^c^SGA was defined as being below the 10th percentile of the national reference. LGA was defined as being above the 10th percentile of the national reference. Denominators are neonatal outcomes without unknown gestational week at delivery, birth weight, sex of neonates, and over 42 weeks at gestation.

CS, cesarean section; SGA, small for gestational age; LGA, large for gestational age.

### Ovarian stimulation protocols and neonatal outcomes

Crude and adjusted ORs of ovarian stimulation protocols for pregnancy and neonatal outcomes are shown in Table 3. Compared with the natural cycle, all ovarian stimulation protocols showed a significantly increased risk for PTD, LBW, and SGA. The CC and CC + gonadotropin protocols showed the highest crude and adjusted odds ratios (ORs) for LBW, VLBW and SGA compared with other protocols. These protocols also exhibited a significantly decreased risk for large for gestational age (LGA), and the CC and CC + gonadotropin protocols were significantly associated with CS.

**Table 3:** Crude and adjusted ORs of ovarian stimulation protocols compared with natural cycle for pregnancy and neonatal outcomes.

Outcomes	Crude OR (95% Cl)	P value	Adjusted OR (95% Cl)^a^	P value
PTD (<37 weeks)				
Natural cycle	Reference		Reference	
Clomiphene alone	1.35 (1.16 to 1.58)	<0.001	1.33 (1.13 to 1.58)	0.001
Clomiphene + gonadotropin	1.33 (1.06 to 1.66)	0.01	1.31 (1.03 to 1.66)	0.03
GnRH agonist	1.39 (1.21 to 1.59)	<0.001	1.34 (1.13 to 1.58)	0.001
GnRH antagonist	1.41 (1.21 to 1.65)	<0.001	1.37 (1.14 to 1.63)	0.001
VPTD (<32 weeks)				
Natural cycle	Reference		Reference	
Clomiphene alone	1.19 (0.83 to 1.71)	0.34	1.16 (0.80 to 1.67)	0.43
Clomiphene + gonadotropin	1.62 (1.06 to 2.48)	0.03	1.61 (1.02 to 2.53)	0.04
GnRH agonist	1.15 (0.81 to 1.63)	0.45	1.13 (0.78 to 1.64)	0.52
GnRH antagonist	1.30 (0.90 to 1.87)	0.16	1.25 (0.85 to 1.83)	0.26
LBW (<2500 g)				
Natural cycle	Reference		Reference	
Clomiphene alone	1.63 (1.48 to 1.79)	<0.001	1.62 (1.46 to 1.79)	<0.001
Clomiphene + gonadotropin	1.67 (1.48 to 1.89)	<0.001	1.67 (1.45 to 1.91)	<0.001
GnRH agonist	1.44 (1.31 to 1.59)	<0.001	1.44 (1.29 to 1.59)	<0.001
GnRH antagonist	1.43 (1.27 to 1.61)	<0.001	1.42 (1.26 to 1.60)	<0.001
VLBW (<1500 g)				
Natural cycle	Reference		Reference	
Clomiphene alone	1.75 (1.27 to 2.41)	0.001	1.69 (1.23 to 2.31)	0.001
Clomiphene + gonadotropin	2.44 (1.60 to 3.72)	<0.001	2.38 (1.52 to 3.72)	<0.001
GnRH agonist	1.47 (1.06 to 2.05)	0.02	1.41 (0.999 to 1.98)	0.051
GnRH antagonist	1.57 (1.06 to 2.30)	0.02	1.47 (0.995 to 2.18)	0.053
SGA^b^				
Natural cycle	Reference		Reference	
Clomiphene alone	1.66 (1.48 to 1.87)	<0.001	1.64 (1.46 to 1.84)	<0.001
Clomiphene + gonadotropin	1.74 (1.50 to 2.02)	<0.001	1.71 (1.47 to 1.98)	<0.001
GnRH agonist	1.25 (1.07 to 1.46)	0.004	1.23 (1.05 to 1.45)	0.01
GnRH antagonist	1.29 (1.07 to 1.54)	0.006	1.27 (1.06 to 1.52)	0.01
LGA^b^				
Natural cycle	Reference		Reference	
Clomiphene alone	0.90 (0.82 to 0.98)	0.01	0.89 (0.80 to 0.99)	0.03
Clomiphene + gonadotropin	0.86 (0.77 to 0.96)	0.01	0.88 (0.78 to 0.998)	0.046
GnRH agonist	0.92 (0.84 to 0.996)	0.04	0.96 (0.88 to 1.05)	0.35
GnRH antagonist	0.96 (0.87 to 1.07)	0.46	0.98 (0.88 to 1.08)	0.66
CS				
Natural cycle	Reference		Reference	
Clomiphene alone	1.22 (1.11 to 1.34)	<0.001	1.18 (1.08 to 1.28)	<0.001
Clomiphene + gonadotropin	1.10 (0.99 to 1.22)	0.08	1.13 (1.004 to 1.26)	0.04
GnRH agonist	0.99 (0.90 to 1.09)	0.82	1.04 (0.96 to 1.13)	0.35
GnRH antagonist	1.07 (0.97 to 1.18)	0.19	1.07 (0.98 to 1.18)	0.14

^a^Adjusted for maternal age, infertility diagnosis, fertilization method, fetal sex and year.

^b^SGA was defined as being below the 10th percentile of the national reference. LGA was defined as being above the 10th percentile of the national reference.

CI, confidence interval; CS, cesarean section; LBW, low birth weight; LGA, large for gestational age; OR, odds ratio; PTD, preterm delivery; SGA, small for gestational age; VLBW, very low birth weight; VPTD, very preterm delivery.

### Subgroup analysis according to different ART treatments

The results of subgroup analysis with a maternal age under 35, luteal support using progesterone, and early cleavage stage ET are shown in Table 4. For PTD, the CC and GnRH antagonist protocols demonstrated a significant association throughout the three-subgroup analysis. Similar significant associations were observed between CC or CC + gonadotropin protocols and LBW, VLBW, SGA and CS. In GnRH agonist and antagonist protocols, significant associations were observed for LBW and for SGA in GnRH antagonist protocol throughout the three-subgroup analysis, but for VLBW, the results were attenuated in some of the subgroup analyses, resulting in non-significant associations.

**Table 4:** Adjusted ORs of ovarian stimulation protocols compared with natural cycle for pregnancy and neonatal outcomes among subgroup of different ART treatment.

Outcomes	Maternal age < 35		Progesterone		Early cleavage ET	
	Adjusted OR (95% Cl)^a^	P value	Adjusted OR (95% Cl)^a^	P value	Adjusted OR (95% Cl)^a^	P value
PTD (<37 weeks)						
Natural cycle	Reference		Reference		Reference	
Clomiphene alone	1.28 (1.04 to 1.58)	0.02	1.27 (1.11 to 1.44)	<0.001	1.29 (1.07 to 1.56)	0.01
Clomiphene + gonadotropin	1.16 (0.90 to 1.50)	0.26	1.22 (0.88 to 1.70)	0.24	1.26 (0.93 to 1.70)	0.13
GnRH agonist	1.21 (0.996 to 1.47)	0.06	1.24 (0.99 to 1.56)	0.06	1.22 (0.99 to 1.48)	0.06
GnRH antagonist	1.35 (1.08 to 1.69)	0.01	1.30 (1.01 to 1.68)	0.045	1.28 (1.02 to 1.60)	0.03
VPTD (<32 weeks)						
Natural cycle	Reference		Reference		Reference	
Clomiphene alone	2.62 (1.62 to 4.26)	<0.001	1.07 (0.76 to 1.52)	0.69	1.23 (0.80 to 1.89)	0.34
Clomiphene + gonadotropin	2.49 (1.12 to 5.51)	0.03	1.52 (0.85 to 2.72)	0.16	1.69 (0.98 to 2.90)	0.06
GnRH agonist	1.75 (0.87 to 3.49)	0.12	1.15 (0.74 to 1.77)	0.53	1.01 (0.63 to 1.62)	0.98
GnRH antagonist	1.92 (0.90 to 4.12)	0.09	1.40 (0.80 to 2.44)	0.24	1.07 (0.67 to 1.70)	0.78
LBW (<2500 g)						
Natural cycle	Reference		Reference		Reference	
Clomiphene alone	1.84 (1.48 to 2.29)	<0.001	1.61 (1.45 to 1.80)	<0.001	1.58 (1.43 to 1.74)	<0.001
Clomiphene + gonadotropin	1.81 (1.49 to 2.20)	<0.001	1.57 (1.30 to 1.90)	<0.001	1.58 (1.36 to 1.83)	<0.001
GnRH agonist	1.50 (1.27 to 1.78)	<0.001	1.28 (1.11 to 1.48)	0.001	1.34 (1.20 to 1.50)	<0.001
GnRH antagonist	1.53 (1.26 to 1.85)	<0.001	1.38 (1.17 to 1.62)	<0.001	1.39 (1.21 to 1.59)	<0.001
VLBW (<1500 g)						
Natural cycle	Reference		Reference		Reference	
Clomiphene alone	3.00 (1.71 to 5.27)	<0.001	1.84 (1.27 to 2.66)	0.001	1.77 (1.29 to 2.44)	<0.001
Clomiphene + gonadotropin	3.38 (1.73 to 6.63)	<0.001	2.61 (1.19 to 5.72)	0.02	2.51 (1.53 to 4.12)	<0.001
GnRH agonist	1.86 (1.04 to 3.32)	0.04	1.73 (1.08 to 2.78)	0.02	1.39 (0.93 to 2.09)	0.11
GnRH antagonist	2.02 (1.03 to 3.96)	0.04	1.95 (1.09 to 3.48)	0.02	1.37 (0.86 to 2.18)	0.19
SGA^b^						
Natural cycle	Reference		Reference		Reference	
Clomiphene alone	1.97 (1.47 to 2.63)	<0.001	1.58 (1.37 to 1.83)	<0.001	1.65 (1.48 to 1.84)	<0.001
Clomiphene + gonadotropin	1.89 (1.48 to 2.41)	<0.001	1.60 (1.31 to 1.95)	<0.001	1.60 (1.37 to 1.88)	<0.001
GnRH agonist	1.35 (1.02 to 1.77)	0.030	1.09 (0.88 to 1.35)	0.44	1.25 (1.04 to 1.50)	0.02
GnRH antagonist	1.41 (1.04 to 1.92)	0.03	1.32 (1.04 to 1.68)	0.03	1.29 (1.03 to 1.62)	0.03
LGA^b^						
Natural cycle	Reference		Reference		Reference	
Clomiphene alone	0.82 (0.71 to 0.94)	0.006	0.88 (0.80 to 0.98)	0.02	0.94 (0.83 to 1.06)	0.32
Clomiphene + gonadotropin	0.78 (0.65 to 0.93)	0.006	0.92 (0.74 to 1.14)	0.44	0.89 (0.76 to 1.04)	0.16
GnRH agonist	0.96 (0.83 to 1.11)	0.60	1.09 (0.95 to 1.24)	0.20	1.01 (0.91 to 1.12)	0.84
GnRH antagonist	0.95 (0.80 to 1.13)	0.54	1.11 (0.94 to 1.32)	0.22	0.996 (0.88 to 1.13)	0.96
CS						
Natural cycle	Reference		Reference		Reference	
Clomiphene alone	1.27 (1.12 to 1.45)	<0.001	1.18 (1.09 to 1.29)	<0.001	1.18 (1.08 to 1.28)	<0.001
Clomiphene + gonadotropin	1.20 (1.01 to 1.43)	0.04	1.10 (0.94 to 1.30)	0.23	1.11 (0.98 to 1.26)	0.09
GnRH agonist	1.06 (0.93 to 1.21)	0.35	1.05 (0.95 to 1.17)	0.33	1.04 (0.95 to 1.14)	0.36
GnRH antagonist	1.05 (0.90 to 1.22)	0.53	1.15 (1.01 to 1.31)	0.03	1.03 (0.93 to 1.14)	0.56

^a^Adjusted for maternal age, infertility diagnosis, fertilization method, fetal sex and year.

^b^SGA was defined as being below the 10th percentile of the national reference. LGA was defined as being above the 10th percentile of the national reference.

CI, confidence interval; CS, cesarean section; LBW, low birth weight; LGA, large for gestational age; OR, odds ratio; PTD, preterm delivery; SGA, small for gestational age; VLBW, very low birth weight; VPTD, very preterm delivery.

### Subgroup analysis restricting the number of oocyte retrievals

Results of the subgroup analysis comparing CC with natural cycle and restricting the number of oocyte retrievals to one are shown in Table 5. Even after restricting the analysis to retrievals that collected a single oocyte, there was a significantly increased risk of PTD, LBW, SGA and CS for ovarian stimulation using CC compared with the natural cycle.

**Table 5:** Crude and adjusted ORs of ovarian stimulation using clomiphene citrate compared with natural cycle for pregnancy and neonatal outcomes among subgroup of one oocyte retrieval.

Outcomes	Cycles with one oocyte retrieval					
	Crude OR (95% Cl)			Adjusted OR (95% Cl)^a^		
	Natural cycle	Clomiphene alone	P value	Natural cycle	Clomiphene alone	P value
**PTD (**<**37 weeks)**	Reference	1.34 (1.09 to 1.65)	0.006	Reference	1.33 (1.06 to 1.66)	0.01
**VPTD (**<**32 weeks)**	Reference	0.91 (0.53 to 1.56)	0.73	Reference	0.85 (0.47 to 1.54)	0.59
**LBW (**<**2500 g)**	Reference	1.50 (1.18 to 1.91)	0.001	Reference	1.44 (1.11 to 1.89)	0.01
**VLBW (**<**1500 g)**	Reference	1.40 (0.87 to 2.27)	0.17	Reference	1.35 (0.79 to 2.30)	0.27
**SGA** ^b^	Reference	1.69 (1.34 to 2.14)	<0.001	Reference	1.68 (1.30 to 2.18)	<0.001
**LGA** ^b^	Reference	1.00 (0.86 to 1.17)	0.97	Reference	0.99 (0.87 to 1.13)	0.93
**CS**	Reference	1.28 (1.17 to 1.41)	<0.001	Reference	1.22 (1.07 to 1.39)	0.003

^a^Adjusted for maternal age, infertility diagnosis, fertilization method, fetal sex and year.

^b^SGA was defined as being below the 10th percentile of the national reference. LGA was defined as being above the 10th percentile of the national reference.

CI, confidence interval; CS, cesarean section; LBW, low birth weight; LGA, large for gestational age; OR, odds ratio; PTD, preterm delivery; SGA, small for gestational age; VLBW, very low birth weight; VPTD, very preterm delivery.

### Sensitivity analysis

Results of the subgroup analysis restricting samples with term deliveries are shown in Supplemental Table 1. Even restricting samples at term deliveries, all the ovarian stimulation protocols were associated with LBW, and significant associations were observed between CC or CC + gonadotropin protocols and SGA, LGA and CS. Further, sensitivity analysis excluding cycles with missing values demonstrated almost the same results, although several significant associations were attenuated and became marginally significant or non-significant (Supplemental Tables 2–4).

## Discussion

Using a nationally-representative ART sample from Japan, we found that ovarian stimulation protocols were significantly associated with lower birth weight compared with natural cycles, even for singleton deliveries following fresh single ET. In particular, ovarian stimulation using CC produced worse neonatal outcomes compared with other stimulation protocols, and was significantly associated with PTD, SGA and CS. Our study suggests that ovarian stimulation may affect birth weight, and CC may have an adverse effect on neonatal outcomes in fresh cycles.

Few studies have investigated the association between ovarian stimulation protocols and neonatal outcomes, and these limited findings have been conflicting. Mak *et al*. recently reported perinatal outcomes among singleton deliveries following natural cycle IVF (n = 190) and stimulated IVF using GnRH agonist or antagonist (n = 174) in a single center between 2007–2013^8^. This recent study suggested that neonates born following natural cycle IVF had a significantly lower risk for LBW (adjusted OR, 0.07, 95% confidence interval [CI], 0.014–0.35). The PTD rates were typically high in both groups, but significantly smaller in natural cycle IVF than in stimulated IVF (31.5% vs. 42.0%, respectively, P = 0.03). However, another study used nationwide U.K. data to investigate perinatal outcomes of singleton births following natural (n = 262) and stimulated IVF cycles (n = 98,667) from 1991–2011. The analysis of U.K. data found ovarian stimulation had no significantly increased risk for LBW (adjusted OR, 1.58, 95% CI, 0.96–2.58) and PTD (adjusted OR, 1.43, 95% CI, 0.91–2.26). Both studies included natural cycle sample sizes that were too small to draw strong conclusions, and did not stratify ovarian stimulation protocols. In Japan, mild ovarian stimulation using CC or natural cycle IVF has been broadly applied in ART institutions^15–17^, resulting in adequate sample numbers, especially for natural cycles, to investigate the association between ovarian stimulation and neonatal outcomes.

Among ovarian stimulation protocols, those using CC demonstrated a higher risk for PTD, LBW, SGA and CS. Similar adverse outcomes following CC have been suggested in non-ART populations. A nationwide retrospective cohort study from Denmark reported that intrauterine insemination with ovulation induction using CC had a significantly increased risk for LBW (adjusted OR, 1.5, 95% CI, 1.1–2.1) and SGA (adjusted OR, 1.6, 95% CI, 1.1–2.4) compared with natural cycle intrauterine insemination^18^. Another study investigating perinatal outcomes of 623 infants born naturally or following CC or letrozole protocols found that birthweight was significantly smaller in the CC group compared with natural (P < 0.02) or letrozole cycles (P < 0.02), even among singletons^19^. These results do not eliminate the possibility that multiple ovulation, resulting in higher serum estradiol levels, may mediate the association between CC and adverse perinatal outcomes^9^. However, our study demonstrated a significant association even after restricting the analysis to one oocyte collected per retrieval cycle, suggesting CC itself may have an adverse effect on perinatal outcomes. CC has both estrogen agonistic and antagonistic properties, which cause depletion of estrogen receptors in the hypothalamus leading to increased GnRH secretion^20^. However, the antiestrogenic effects of CC on the endometrium, implantation and subsequent gestation remain unknown. One study reported that although more than 85% of CC was eliminated in approximately 6 days, significant plasma concentrations of the Z-isomer of CC was detected 1 month after administration^21^. Other research suggested that CC may suppress endometrium receptivity^22,23^ or cause morphological changes in the endometrium^24,25^. An ovarian stimulation protocol administering CC during the whole stimulation phase was reported to prevent the premature surge of luteinizing hormone^15,26^. For such cases, the negative effect of CC may be strengthened compared with the normal shorter dosage regime for ovulation induction.

One strength of the current study is that we restricted our analysis to singleton deliveries following fresh single ET from ovulatory women to eliminate the influence of multiple pregnancies, vanishing twins and PCOS on neonatal outcomes. After the introduction of the SET policy in 2007, single ET now represents more than 70% of all ETs in Japan^27^, resulting in improvements in perinatal outcomes. However, there are several limitations in our study. First, specific indicators for selecting an ovarian stimulation protocol were unavailable, which may give rise to the possibility of residual confounding effects from underlying indicator factors. Second, we lacked data on important confounders such as parity, duration of infertility, numbers of previous ART failures, maternal body mass index and smoking status, which may also confound the findings. Third, other mediating factors such as embryo quality may play a role in the association between ovarian stimulation protocols and neonatal outcomes. Finally, the registry consists of cycle-specific information, and it is not possible to adjust for correlations if women had multiple deliveries during the study period. However, since Japan has one of the lowest birth rates in the world (total fertility rate of 1.5 in 2015)^28^, the number of women who had multiple deliveries between 2007 and 2013 would be small. Based on the above limitations, further studies, especially randomized controlled trials investigating the effect of ovarian stimulation protocols upon neonatal outcomes, are essential.

Although it has been reported that perinatal outcomes of fresh ET cycles tend to be worse compared with those of frozen cycles, even for singletons, our study suggests that ovarian stimulation protocols play an important role in birth weight and gestational length in fresh cycles. Considering that the endometrium can be affected by ovarian stimulation^29^, and the improvements in vitrification, it is possible that a frozen ET may provide a better option instead of fresh ET following ovarian stimulation, in order to achieve better perinatal outcomes.

In conclusion, using a nationally-representative Japanese ART sample, we found that ovarian stimulation was significantly associated with lower birthweight after fresh cycles. In particular, the use of CC in ovarian stimulation had a higher risk of adverse perinatal outcomes compared with other stimulation protocols, and was significantly associated with PTD, SGA and CS. Considering our current findings, frozen ET may be an alternative option from the perspective of perinatal outcomes. Further studies, especially randomized controlled trials, are needed to investigate the effect of ovarian stimulation using CC on endometrium, implantation and subsequent gestation.

## Methods

### Study sample

This is a retrospective cohort study using a Japanese national ART registry assembled by the Japan Society of Obstetrics and Gynecology (JSOG). The JSOG launched the ongoing registration system in 2007 for all ART clinics and hospitals to report cycle-specific information on-line. The registry has mandatory reporting, and patients cannot receive government subsidies if a clinic or hospital does not register their information. The database included cycle-specific information such as infertility diagnosis, ovarian stimulation protocols, IVF or intracytoplasmic sperm injection (ICSI), embryo stage at transfer, and pregnancy and obstetric outcomes. The JSOG requires all participating clinics and hospitals to report pregnancy and obstetric outcomes. ART clinics without delivery facilities usually receive a hospital delivery report, and if they do not obtain the delivery report, the JSOG recommends ART facilities contact mothers directly to obtain obstetrical outcomes. Since the use of donor oocytes or embryos is prohibited during ART in Japan, all embryos transferred were autologous. Preimplantation genetic testing for chromosomal aneuploidy is prohibited in Japan.

**Figure 1:**
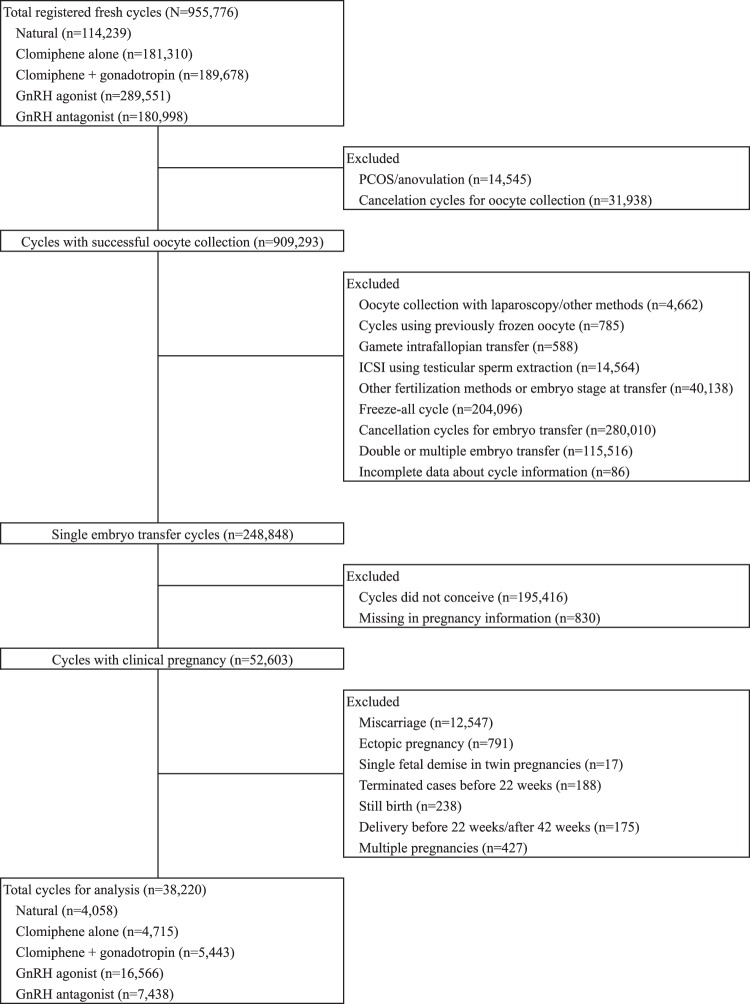
Flow diagram of cohort selection and comparison groups.

We included singleton live births after 22 weeks of gestation, or birth weight > 500 g with unknown gestational length, following fresh single ETs between 2007 and 2013. We excluded cycles with polycystic ovary syndrome or anovulation, ICSI using testicular sperm extraction, and gamete intra-fallopian transfers. A detailed flow diagram of the cohort selection process is shown in Fig. 1. Among 248,848 single embryo transfer cycles, 52,603 cycles resulted in clinical pregnancy. After excluding cycles with miscarriages, ectopic pregnancies, single fetal demise in twin pregnancies, terminated cases, still births, delivery before 22/after 42 weeks and multiple pregnancies, 38,220 cases were included in this study.

### Ethical approval

This study was approved by the institutional review board at the National Center for Child Health and Development, Saitama Medical University and ethics committee of the JSOG. After approval of the study, the JSOG provided data without any personal identifying information. The study was conducted in accordance with Japanese law and the STROBE Guidelines. No informed consent was obtained from the patients because the study was retrospective.

### Outcomes examined

Our main outcomes were birth weight and gestational length. LBW was defined as birth weight less than 2500 g. VLBW was defined as birth weight less than 1500 g. PTD was defined as gestational weeks at delivery less than 37 weeks, VPTD was defined as gestational weeks at delivery less than 32 weeks. Similarly, SGA and LGA were defined below/above the 10^th^ percentile for neonates born between 22 and 41 weeks according to the national reference^30^. We also investigated delivery methods of CS as a secondary outcome.

### Other variables

Ovarian stimulation protocols included natural (i.e., unstimulated), CC alone, CC with gonadotropin (CC + gonadotropin), GnRH agonist and GnRH antagonist protocols. We also used maternal age, infertility diagnosis, number of oocytes retrieved, fertilization method (IVF, ICSI or split-ICSI) and embryo stage at transfer (early cleavage or blastocyst).

### Statistical analysis

We compared baseline characteristics and perinatal outcomes according to ovarian stimulation protocols using the χ^2^ test or one-way analysis of variance. We calculated the crude and adjusted OR of each ovarian stimulation protocol compared with natural cycles for neonatal outcomes using generalized estimating equations with robust variance estimation adjusting for correlations within ART institutions. The *a priori* covariates for adjusted analysis were maternal age (categorized into 5-year age groups), infertility diagnosis, fertilization method (i.e. IVF/ICSI), fetal sex and reported year of cycles. Since we included cycles with incomplete data about obstetric outcomes, there were missing values in delivery method (8.7%), gestational age at delivery (8.8%), birth weight (7.7%) and sex of neonates (7.5%). For those variables, we performed multiple imputation by chained equations to impute missing data with 10 sets of imputations, and then conducted regression analysis. Further, we conducted subgroup analysis with maternal age under 35 years to exclude the effect of advanced maternal age on perinatal outcomes. Since luteal support and embryo stage at transfer are mediating factors between ovarian stimulation and perinatal outcomes, and adjusting for those variables is not appropriate^31^, we conducted subgroup analysis restricting luteal support to progesterone alone, or cycles with early cleavage ETs. Finally, in order to remove the effect of multiple oocytes collected in a single retrieval on outcomes, we compared neonatal outcomes following ovarian stimulation using CC alone with a natural cycle from ART cycles with just one oocyte retrieved.

We conducted two sensitivity analyses. The first analysis was restricting samples within term deliveries (gestational age at delivery between 37 and 41 weeks of gestation). Second, we performed all analysis with complete-case analysis (i.e. excluding cycles with missing values). All analyses were performed using the STATA SE statistical package, version 13.1 (Stata, College Station, TX, USA). A two-tailed value of P < 0.05 was considered statistically significant.

## Supplementary information

Supplemental tables

## Data Availability

The datasets analyzed during the current study are not publicly available since the datasets include special care-required personal information but are available from the corresponding author on reasonable request.
