# Supplementary material for: Neonatal outcomes following different ovarian stimulation protocols in fresh single embryo transfer
Source: Sci Rep. 2019 Feb 28;9:3076. doi: 10.1038/s41598-019-38724-2 (PMC6395801; doi:10.1038/s41598-019-38724-2)
Supplement: Supplementary file 1 — Supplemental tables [file 41598_2019_38724_MOESM1_ESM.docx]

**Neonatal outcomes following different ovarian stimulation protocols in single fresh embryo transfer**

Seung Chik Jwa^1, 2*^, Akira Nakashima^2^, Akira Kuwahara^3^, Kazuki Saito^4^, Minoru Irahara^3^, Tetsuro Sakumoto^2^, Osamu Ishihara^1^, Hidekazu Saito^5^

^1^Department of Obstetrics and Gynecology, Saitama Medical University, Saitama, Japan.

^2^Sora no Mori Clinic, Okinawa, Japan.

^3^Department of Obstetrics and Gynecology, Graduate School of Biomedical Sciences, Tokushima University, Tokushima, Japan.

^4^Department of Comprehensive Reproductive Medicine, Graduate School, Tokyo Medical and Dental University, Tokyo, Japan.

^5^Division of Reproductive Medicine, Center of Maternal–Fetal, Neonatal and Reproductive Medicine, National Center for Child Health and Development, Tokyo, Japan.

***Corresponding author** **to whom reprint requests should be addressed**:

Department of Obstetrics and Gynecology, Saitama Medical University, 38 Morohongo, Moroyama, Saitama, 350-0495, JAPAN.

Tel: +81-49-276-1347; fax: +81-49-294-8305; e-mail: jwa_s@saitama-med.ac.jp

**Supplemental table legends:**

**Supplemental Table 1.** Crude and adjusted ORs of ovarian stimulation protocols compared with natural cycle for pregnancy and neonatal outcomes among subgroup of term deliveries.

**Supplemental Table 2.** Crude and adjusted ORs of ovarian stimulation protocols compared with natural cycle for pregnancy and neonatal outcomes excluding cycles with missing values.

**Supplemental Table 3.** Adjusted ORs of ovarian stimulation protocols compared with natural cycle for pregnancy and neonatal outcomes among subgroup of different ART treatment excluding cycles with missing values.

**Supplemental Table 4.** Crude and adjusted ORs of ovarian stimulation using clomiphene citrate compared with natural cycle for pregnancy and neonatal outcomes among subgroup of one oocyte retrieval excluding cycles with missing values.

| Supplemental Table 1. Crude and adjusted ORs of ovarian stimulation protocols compared with natural cycle for pregnancy and neonatal outcomes among subgroup of term deliveries. | | | | |
| --- | --- | --- | --- | --- |
| Outcomes | Gestational age at delivery ≥ 37 | | | |
|  | Crude OR (95% Cl) ^a^ | P value | Adjusted OR (95% Cl) ^a^ | P value |
| **LBW (<2500 g)** |  |  |  |  |
| Natural cycle | Reference |  | Reference |  |
| Clomiphene alone | 1.64 (1.37 to 1.96) | <0.001 | 1.65 (1.40 to 1.94) | <0.001 |
| Clomiphene + gonadotropin | 1.71 (1.39 to 2.10) | <0.001 | 1.72 (1.42 to 2.08) | <0.001 |
| GnRH agonist | 1.47 (1.20 to 1.81) | <0.001 | 1.51 (1.24 to 1.83) | <0.001 |
| GnRH antagonist | 1.46 (1.17 to 1.82) | 0.001 | 1.48 (1.21 to 1.82) | <0.001 |
| **VLBW (<1500 g)** |  |  |  |  |
| Natural cycle | Reference |  | Reference |  |
| Clomiphene alone | 0.69 (0.11 to 4.34) | 0.69 | 0.52 (0.08 to 3.20) | 0.48 |
| Clomiphene + gonadotropin | 1.51 (0.30 to 7.46) | 0.61 | 1.34 (0.26 to 6.84) | 0.72 |
| GnRH agonist | 1.79 (0.34 to 9.34) | 0.49 | 1.08 (0.21 to 5.63) | 0.93 |
| GnRH antagonist | 1.31 (0.25 to 6.79) | 0.75 | 1.01 (0.12 to 8.34) | 0.99 |
| **SGA**^b^ |  |  |  |  |
| Natural cycle | Reference |  | Reference |  |
| Clomiphene alone | 1.48 (1.26 to 1.74) | <0.001 | 1.49 (1.26 to 1.72) | <0.001 |
| Clomiphene + gonadotropin | 1.54 (1.25 to 1.90) | <0.001 | 1.50 (1.23 to 1.82) | <0.001 |
| GnRH agonist | 1.17 (0.96 to 1.43) | 0.12 | 1.17 (0.95 to 1.43) | 0.14 |
| GnRH antagonist | 1.23 (0.99 to 1.54) | 0.06 | 1.23 (0.98 to 1.54) | 0.07 |
| **LGA**^b^ |  |  |  |  |
| Natural cycle |  |  |  |  |
| Clomiphene alone | 0.91 (0.84 to 0.99) | 0.04 | 0.90 (0.81 to 0.99) | 0.04 |
| Clomiphene + gonadotropin | 0.86 (0.74 to 0.99) | 0.03 | 0.85 (0.73 to 0.99) | 0.049 |
| GnRH agonist | 0.91 (0.82 to 0.998) | 0.047 | 0.94 (0.85 to 1.03) | 0.20 |
| GnRH antagonist | 0.95 (0.85 to 1.06) | 0.35 | 0.99 (0.85 to 1.06) | 0.35 |
| **CS** |  |  |  |  |
| Natural cycle |  |  |  |  |
| Clomiphene alone | 1.14 (1.04 to 1.26) | 0.01 | 1.12 (1.02 to 1.23) | 0.02 |
| Clomiphene + gonadotropin | 1.03 (0.93 to 1.14) | 0.61 | 1.06 (0.96 to 1.18) | 0.26 |
| GnRH agonist | 0.95 (0.86 to 1.05) | 0.34 | 1.02 (0.94 to 1.11) | 0.60 |
| GnRH antagonist | 1.03 (0.92 to 1.15) | 0.62 | 1.05 (0.95 to 1.16) | 0.32 |
| ^a^ Adjusted for maternal age, infertility diagnosis, fertilization method, fetal sex and year. | | | | |
| ^b^ SGA was defined as being below the 10th percentile of the national reference. LGA was defined as being above the 10th percentile of the national reference. | | | | |
| CI, confidence interval; CS, cesarean section; LBW, low birth weight; LGA, large for gestational age; OR, odds ratio; SGA, small for gestational age; VLBW, very low birth weight. | | | | |

| Supplemental Table 2. Crude and adjusted ORs of ovarian stimulation protocols compared with natural cycle for pregnancy and neonatal outcomes excluding cycles with missing values. | | | | |
| --- | --- | --- | --- | --- |
| Outcomes | Crude OR (95% Cl) | P value | Adjusted OR (95% Cl) ^a^ | P value |
| **PTD (<37 weeks)** |  |  |  |  |
| Natural cycle | Reference |  | Reference |  |
| Clomiphene alone | 1.36 (1.16 to 1.58) | <0.001 | 1.34 (1.12 to 1.61) | 0.001 |
| Clomiphene + gonadotropin | 1.34 (1.05 to 1.71) | 0.02 | 1.29 (0.99 to 1.68) | 0.06 |
| GnRH agonist | 1.39 (1.21 to 1.59) | <0.001 | 1.33 (1.11 to 1.58) | 0.002 |
| GnRH antagonist | 1.41 (1.21 to 1.65) | <0.001 | 1.35 (1.12 to 1.64) | 0.002 |
| **VPTD (<32 weeks)** |  |  |  |  |
| Natural cycle | Reference |  | Reference |  |
| Clomiphene alone | 1.19 (0.85 to 1.67) | 0.31 | 1.22 (0.82 to 1.82) | 0.32 |
| Clomiphene + gonadotropin | 1.67 (1.08 to 2.57) | 0.02 | 1.63 (0.97 to 2.74) | 0.06 |
| GnRH agonist | 1.12 (0.80 to 1.57) | 0.51 | 1.09 (0.72 to 1.67) | 0.68 |
| GnRH antagonist | 1.27 (0.89 to 1.82) | 0.19 | 1.25 (0.81 to 1.91) | 0.32 |
| **LBW (<2500 g)** |  |  |  |  |
| Natural cycle | Reference |  | Reference |  |
| Clomiphene alone | 1.65 (1.50 to 1.82) | <0.001 | 1.65 (1.47 to 1.85) | <0.001 |
| Clomiphene + gonadotropin | 1.71 (1.50 to 1.95) | <0.001 | 1.69 (1.45 to 1.97) | <0.001 |
| GnRH agonist | 1.45 (1.31 to 1.60) | <0.001 | 1.44 (1.29 to 1.62) | <0.001 |
| GnRH antagonist | 1.45 (1.28 to 1.63) | <0.001 | 1.43 (1.26 to 1.63) | <0.001 |
| **VLBW (<1500 g)** |  |  |  |  |
| Natural cycle | Reference |  | Reference |  |
| Clomiphene alone | 1.85 (1.31 to 2.60) | <0.001 | 1.85 (1.27 to 2.69) | 0.001 |
| Clomiphene + gonadotropin | 2.65 (1.64 to 4.27) | <0.001 | 2.59 (1.50 to 4.47) | 0.001 |
| GnRH agonist | 1.55 (1.09 to 2.18) | 0.01 | 1.48 (0.99 to 2.22) | 0.06 |
| GnRH antagonist | 1.60 (1.10 to 2.35) | 0.02 | 1.56 (1.02 to 2.37) | 0.04 |
| **SGA**^b^ |  |  |  |  |
| Natural cycle | Reference |  | Reference |  |
| Clomiphene alone | 1.69 (1.52 to 1.88) | <0.001 | 1.67 (1.49 to 1.86) | <0.001 |
| Clomiphene + gonadotropin | 1.83 (1.59 to 2.11) | <0.001 | 1.80 (1.56 to 2.07) | <0.001 |
| GnRH agonist | 1.26 (1.09 to 1.46) | 0.002 | 1.24 (1.06 to 1.44) | 0.007 |
| GnRH antagonist | 1.31 (1.10 to 1.55) | 0.002 | 1.29 (1.09 to 1.53) | 0.003 |
| **LGA**^b^ |  |  |  |  |
| Natural cycle | Reference |  | Reference |  |
| Clomiphene alone | 0.86 (0.82 to 0.95) | 0.001 | 0.88 (0.81 to 0.97) | 0.007 |
| Clomiphene + gonadotropin | 0.87 (0.78 to 0.97) | 0.02 | 0.88 (0.78 to 0.99) | 0.04 |
| GnRH agonist | 0.91 (0.84 to 0.99) | 0.03 | 0.96 (0.88 to 1.05) | 0.36 |
| GnRH antagonist | 0.95 (0.86 to 1.05) | 0.34 | 0.97 (0.88 to 1.07) | 0.51 |
| **CS** |  |  |  |  |
| Natural cycle | Reference |  | Reference |  |
| Clomiphene alone | 1.23 (1.12 to 1.34) | <0.001 | 1.19 (1.10 to 1.29) | <0.001 |
| Clomiphene + gonadotropin | 1.11 (0.99 to 1.23) | 0.06 | 1.14 (1.01 to 1.29) | 0.04 |
| GnRH agonist | 0.99 (0.90 to 1.09) | 0.81 | 1.04 (0.96 to 1.13) | 0.33 |
| GnRH antagonist | 1.07 (0.97 to 1.18) | 0.18 | 1.08 (0.99 to 1.18) | 0.09 |
| ^a^ Adjusted for maternal age, infertility diagnosis, fertilization method, fetal sex and year. | | | |  |
| ^b^ SGA was defined as being below the 10th percentile of the national reference. LGA was defined as being above the 10th percentile of the national reference. | | | | |
| CI, confidence interval; CS, cesarean section; LBW, low birth weight; LGA, large for gestational age; OR, odds ratio; PTD, preterm delivery; SGA, small for gestational age; VLBW, very low birth weight; VPTD, very preterm delivery. | | | | |

| Supplemental table 3. Adjusted ORs of ovarian stimulation protocols compared with natural cycle for pregnancy and neonatal outcomes among subgroup of different ART treatment excluding cycles with missing values. | | | | | | |  |
| --- | --- | --- | --- | --- | --- | --- | --- |
| Outcomes | Maternal age <35 | | Progesterone alone | | Early cleavage ET | |  |
|  | Adjusted OR (95% Cl) ^a^ | P value | Adjusted OR (95% Cl) ^a^ | P value | Adjusted OR (95% Cl) ^a^ | P value |  |
| **PTD (<37 weeks)** |  |  |  |  |  |  |  |
| Natural cycle | Reference |  | Reference |  | Reference |  |  |
| Clomiphene alone | 1.29 (1.07 to 1.57) | 0.01 | 1.28 (1.12 to 1.47) | <0.001 | 1.30 (1.07 to 1.58) | 0.01 |  |
| Clomiphene + gonadotropin | 1.15 (0.89 to 1.50) | 0.29 | 1.21 (0.86 to 1.71) | 0.27 | 1.22 (0.89 to 1.68) | 0.14 |  |
| GnRH agonist | 1.20 (0.98 to 1.46) | 0.07 | 1.24 (0.986 to 1.57) | 0.07 | 1.20 (0.97 to 1.47) | 0.09 |  |
| GnRH antagonist | 1.33 (1.06 to 1.67) | 0.01 | 1.26 (0.96 to 1.64) | 0.10 | 1.26 (0.99 to 1.61) | 0.054 |  |
| **VPTD (<32 weeks)** |  |  |  |  |  |  |  |
| Natural cycle | Reference |  | Reference |  | Reference |  |  |
| Clomiphene alone | 3.12 (1.73 to 5.63) | <0.001 | 1.00 (0.80 to 1.52) | 0.54 | 1.25 (0.80 to 1.98) | 0.33 |  |
| Clomiphene + gonadotropin | 2.71 (1.01 to 7.28) | 0.048 | 1.52 (0.80 to 2.88) | 0.20 | 1.63 (0.91 to 2.93) | 0.10 |  |
| GnRH agonist | 1.87 (0.75 to 4.68) | 0.18 | 1.13 (0.72 to 1.78) | 0.59 | 0.93 (0.56 to 1.55) | 0.78 |  |
| GnRH antagonist | 2.13 (0.79 to 5.71) | 0.13 | 1.40 (0.79 to 2.48) | 0.24 | 1.04 (0.63 to 1.71) | 0.87 |  |
| **LBW (<2500 g)** |  |  |  |  |  |  |  |
| Natural cycle | Reference |  | Reference |  | Reference |  |  |
| Clomiphene alone | 1.91 (1.56 to 2.33) | <0.001 | 1.63 (1.45 to 1.83) | <0.001 | 1.60 (1.43 to 1.79) | <0.001 |  |
| Clomiphene + gonadotropin | 1.86 (1.49 to 2.31) | <0.001 | 1.60 (1.29 to 1.99) | <0.001 | 1.58 (1.34 to 1.85) | <0.001 |  |
| GnRH agonist | 1.52 (1.29 to 1.81) | <0.001 | 1.27 (1.09 to 1.48) | 0.002 | 1.33 (1.19 to 1.50) | <0.001 |  |
| GnRH antagonist | 1.56 (1.28 to 1.89) | <0.001 | 1.39 (1.17 to 1.66) | <0.001 | 1.39 (1.20 to 1.61) | <0.001 |  |
| **VLBW (<1500 g)** |  |  |  |  |  |  |  |
| Natural cycle | Reference |  | Reference |  | Reference |  |  |
| Clomiphene alone | 3.77 (2.27 to 6.27) | <0.001 | 1.91 (1.27 to 2.88) | 0.002 | 1.97 (1.32 to 2.94) | 0.001 |  |
| Clomiphene + gonadotropin | 4.31 (1.84 to 10.1) | 0.001 | 2.72 (1.14 to 6.48) | 0.02 | 2.72 (1.44 to 5.14) | 0.002 |  |
| GnRH agonist | 2.24 (1.11 to 4.54) | 0.03 | 1.76 (1.08 to 2.87) | 0.02 | 1.47 (0.90 to 2.39) | 0.12 |  |
| GnRH antagonist | 2.46 (1.14 to 5.34) | 0.02 | 1.92 (1.05 to 3.51) | 0.03 | 1.45 (0.89 to 2.35) | 0.14 |  |
| **SGA**^b^ |  |  |  |  |  |  |  |
| Natural cycle | Reference |  | Reference |  | Reference |  |  |
| Clomiphene alone | 2.01 (1.52 to 2.66) | <0.001 | 1.60 (1.40 to 1.83) | <0.001 | 1.67 (1.51 to 1.84) | <0.001 |  |
| Clomiphene + gonadotropin | 2.00 (1.58 to 2.53) | <0.001 | 1.65 (1.37 to 1.99) | <0.001 | 1.66 (1.43 to 1.92) | <0.001 |  |
| GnRH agonist | 1.37 (1.05 to 1.78) | 0.02 | 1.08 (0.88 to 1.33) | 0.45 | 1.25 (1.05 to 1.49) | 0.01 |  |
| GnRH antagonist | 1.44 (1.08 to 1.93) | 0.01 | 1.36 (1.07 to 1.71) | 0.01 | 1.31 (1.06 to 1.62) | 0.01 |  |
| **LGA**^b^ |  |  |  |  |  |  |  |
| Natural cycle | Reference |  | Reference |  | Reference |  |  |
| Clomiphene alone | 0.81 (0.72 to 0.90) | <0.001 | 0.88 (0.80 to 0.97) | 0.01 | 0.93 (0.83 to 1.04) | 0.21 |  |
| Clomiphene + gonadotropin | 0.77 (0.65 to 0.91) | 0.002 | 0.92 (0.74 to 1.14) | 0.45 | 0.89 (0.77 to 1.04) | 0.16 |  |
| GnRH agonist | 0.97 (0.85 to 1.11) | 0.66 | 1.09 (0.96 to 1.25) | 0.20 | 1.02 (0.92 to 1.13) | 0.73 |  |
| GnRH antagonist | 0.95 (0.80 to 1.12) | 0.52 | 1.11 (0.95 to 1.29) | 0.20 | 0.99 (0.87 to 1.12) | 0.82 |  |
| **CS** |  |  |  |  |  |  |  |
| Natural cycle | Reference |  | Reference |  | Reference |  |  |
| Clomiphene alone | 1.28 (1.14 to 1.43) | <0.001 | 1.20 (1.10 to 1.30) | <0.001 | 1.19 (1.10 to 1.28) | <0.001 |  |
| Clomiphene + gonadotropin | 1.21 (1.03 to 1.42) | 0.02 | 1.12 (0.94 to 1.35) | 0.21 | 1.12 (0.97 to 1.28) | 0.11 |  |
| GnRH agonist | 1.05 (0.94 to 1.18) | 0.37 | 1.04 (0.94 to 1.16) | 0.42 | 1.04 (0.95 to 1.14) | 0.35 |  |
| GnRH antagonist | 1.04 (0.90 to 1.19) | 0.62 | 1.14 (1.01 to 1.29) | 0.04 | 1.03 (0.93 to 1.15) | 0.52 |  |
| ^a^ Adjusted for maternal age, infertility diagnosis, fertilization method, fetal sex and year. | | | |  |  |  |  |
| ^b^ SGA was defined as being below the 10th percentile of the national reference. LGA was defined as being above the 10th percentile of the national reference. | | | | | | | |
| CI, confidence interval; CS, cesarean section; LBW, low birth weight; LGA, large for gestational age; OR, odds ratio; PTD, preterm delivery; SGA, small for gestational age; VLBW, very low birth weight; VPTD, very preterm delivery. | | | | | | | |

| Supplemental table 4. Crude and adjusted ORs of ovarian stimulation using clomiphene citrate compared with natural cycle for pregnancy and neonatal outcomes among subgroup of one oocyte retrieval excluding cycles with missing values. | | | | | | | |
| --- | --- | --- | --- | --- | --- | --- | --- |
| Outcomes | Cycles with one oocyte retrieval | | | | | | |
|  | Crude OR (95% Cl) | | |  | Adjusted OR (95% Cl) ^a^ | | |
|  | Natural cycle | Clomiphene alone | P value |  | Natural cycle | Clomiphene alone | P value |
| **PTD (<37 weeks)** | Reference | 1.35 (1.10 to 1.65) | 0.004 |  | Reference | 1.34 (1.07 to 1.68) | 0.01 |
| **VPTD (<32 weeks)** | Reference | 0.88 (0.55 to 1.40) | 0.59 |  | Reference | 0.85 (0.47 to 1.54) | 0.59 |
| **LBW (<2500 g)** | Reference | 1.52 (1.17 to 1.96) | 0.001 |  | Reference | 1.47 (1.10 to 1.95) | 0.01 |
| **VLBW (<1500 g)** | Reference | 1.46 (0.87 to 2.45) | 0.16 |  | Reference | 1.47 (0.77 to 2.79) | 0.24 |
| **SGA**^b^ | Reference | 1.72 (1.35 to 2.21) | <0.001 |  | Reference | 1.71 (1.30 to 2.24) | <0.001 |
| **LGA**^b^ | Reference | 0.99 (0.86 to 1.16) | 0.99 |  | Reference | 0.99 (0.88 to 1.12) | 0.90 |
| **CS** | Reference | 1.29 (1.18 to 1.40) | <0.001 |  | Reference | 1.23 (1.08 to 1.39) | 0.001 |
| ^a^ Adjusted for maternal age, infertility diagnosis, fertilization method, fetal sex and year. | | | | | |  |  |
| ^b^ SGA was defined as being below the 10th percentile of the national reference. LGA was defined as being above the 10th percentile of the national reference. | | | | | | | |
| CI, confidence interval; CS, cesarean section; LBW, low birth weight; LGA, large for gestational age; OR, odds ratio; PTD, preterm delivery; SGA, small for gestational age; VLBW, very low birth weight; VPTD, very preterm delivery. | | | | | | | |
